# Supplementary material for: Pseudoknot length modulates the folding, conformational dynamics, and robustness of Xrn1 resistance of flaviviral xrRNAs
Source: Nat Commun. 2021 Nov 5;12:6417. doi: 10.1038/s41467-021-26616-x (PMC8571300; doi:10.1038/s41467-021-26616-x)
Supplement: Supplementary file 1 — Supplementary Information [file 41467_2021_26616_MOESM1_ESM.pdf]

Supporting Information

Pseudoknot length modulates the folding, conformational dynamics and robustness of Xrn1 resistance of flaviviral xrRNAs

Xiaolin Niu[1, 5], Ruirui Sun[1, 2, 5], Zhifeng Chen[1, 3], Yirong Yao[1, 2], Xiaobing Zuo[4], Chunlai Chen[1, 2, *], Xianyang Fang[1, *]

[1]Beijing Advanced Innovation Center for Structural Biology, School of Life Sciences, Tsinghua University, Beijing 100084, China. [2]Beijing Frontier Research Center for Biological Structure, School of Life Sciences, Tsinghua University, Beijing 100084, China. [3]State Key Laboratory for the Chemistry and Molecular Engineering of Medicinal Resources, School of Chemistry and Pharmaceutical Sciences, Guangxi Normal University, Guilin 541004, China. [4]X-ray Science Division, Argonne National Laboratory, Lemont IL 60439, USA.

[5]These authors contributed equally: Xiaolin Niu, Ruirui Sun.

*e-mail: chunlai@mail.tsinghua.edu.cn; fangxy@tsinghua.edu.cn

Table of Contents

Supplementary Figure S1 3
Supplementary Figure S2 5
Supplementary Figure S3 7
Supplementary Figure S4 8
Supplementary Figure S5 9
Supplementary Figure S6 10
Supplementary Figure S7 11
Supplementary Figure S8 12
Supplementary Figure S9 13
Supplementary Table S1 14
Supplementary Table S2 15
Supplementary Table S3 16
Supplementary Table S4 18
Supplementary Table S5 20
References 21

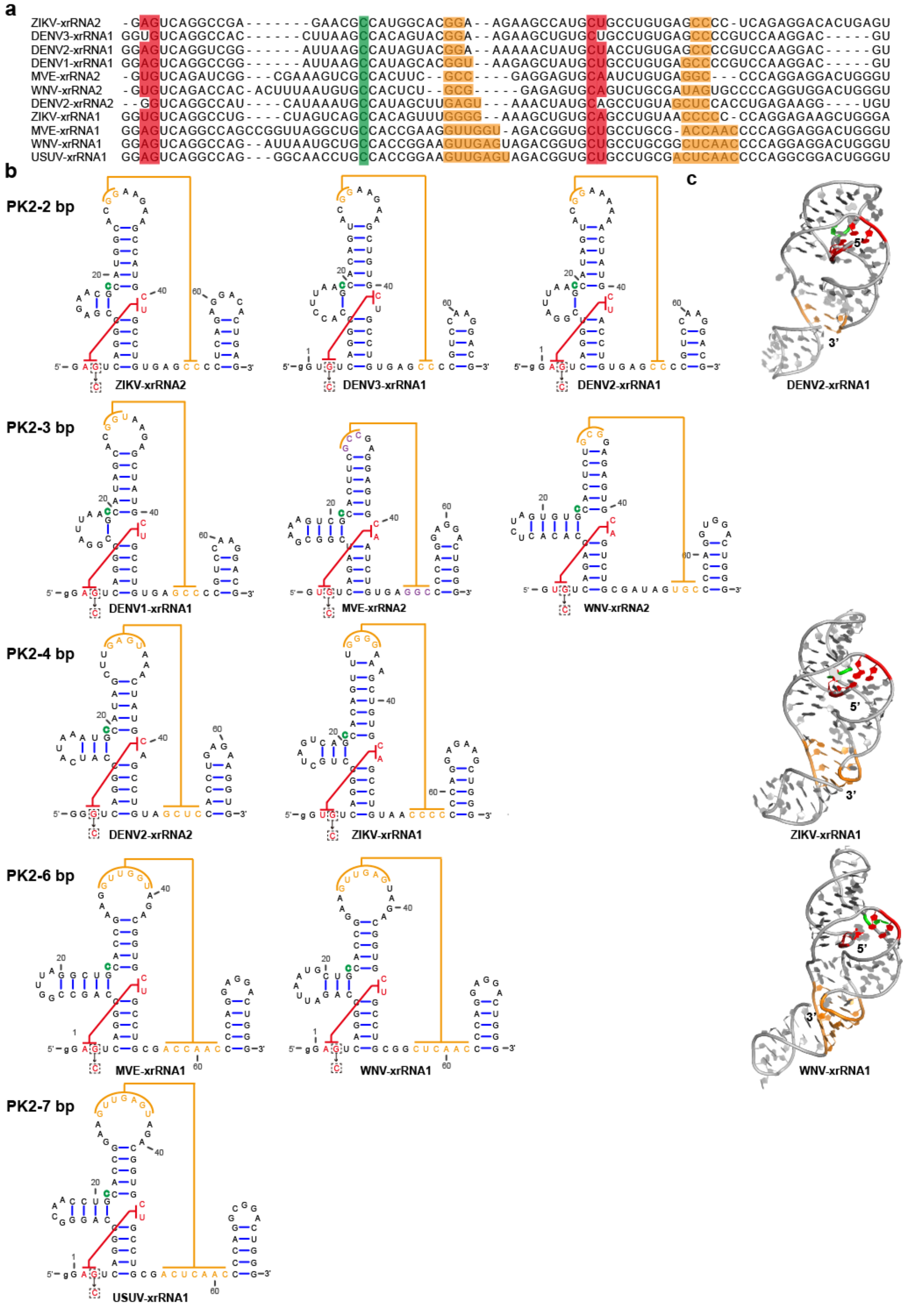

Figure S1. **The primary sequences, secondary and tertiary structures are highly conserved among flaviviral subclass 1a xrRNAs. (a)** Sequence alignment of the 11 flaviviral subclass 1a xrRNAs used in this study. Nucleotides colored in red and orange are involved in the formation of long-range pseudoknot PK1 and PK2 interactions, respectively. The conserved nucleotide C between P2 and P3 (J2/3) is highlighted in

green. (**b**) Proposed secondary structures of 11 flaviviral xrRNAs. The PK1 (G3C) mutants of all xrRNAs used in this study are indicated with boxes. The PK2 lengths of the xrRNAs are indicated on the left. (**c**) Using the crystal structure of ZIKV-xrRNA1 (PDB ID: 5TPY) as template[1], the homology 3D structure models of DENV2-xrRNA1 and WNV-xrRNA1 were built up using ModeRNA[2]. The conserved pseudoknot interactions (PK1 and PK2) and J2/3 nucleotides in the models are colored as that in **a**.

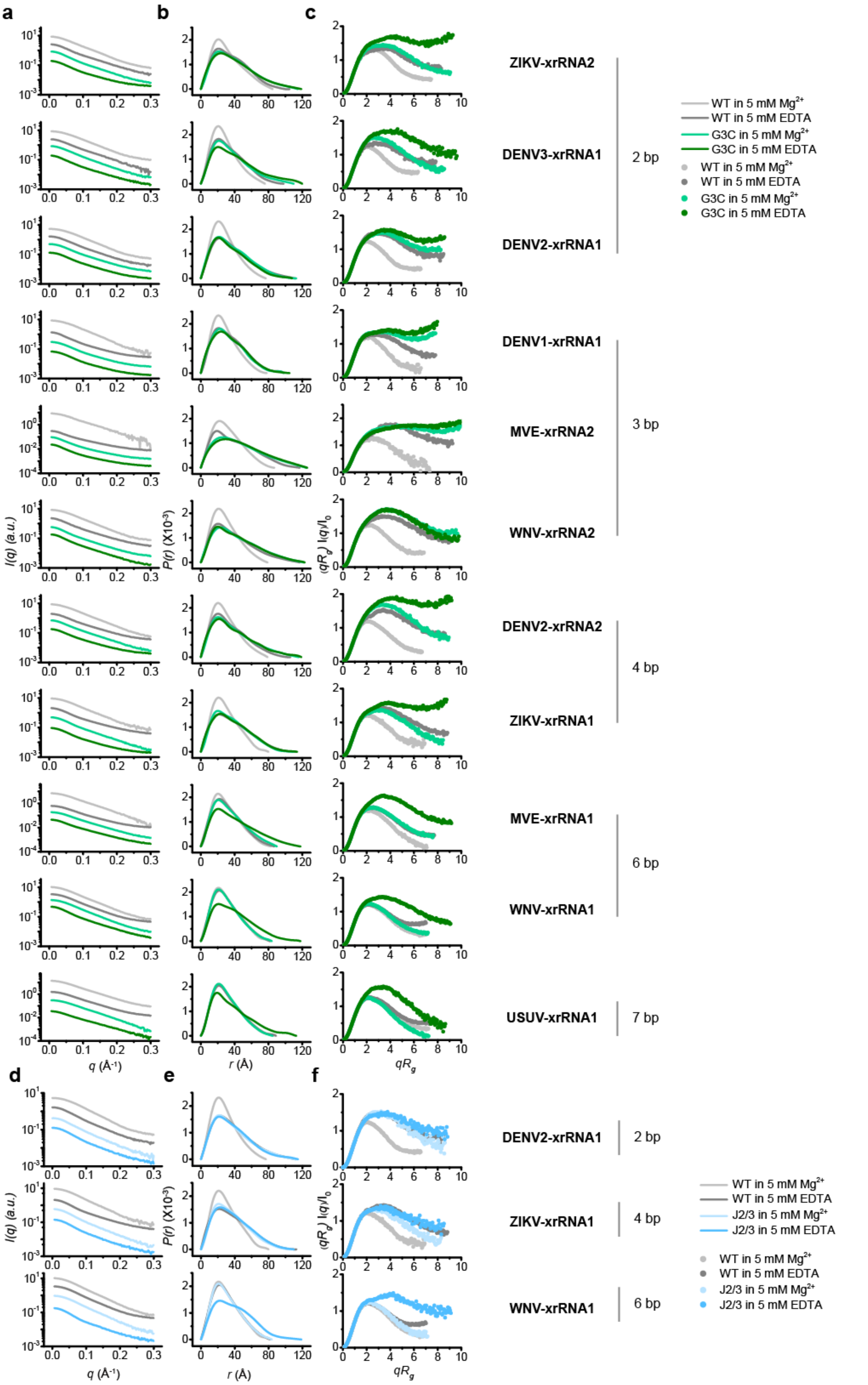

Figure S2. **SAXS analysis of the effects of Mg^{2+} and mutations at key tertiary interaction motifs on the folding of flaviviral xrRNAs. (a-c)** The scattering curves **(a),** normalized PDDFs (**b**) and the dimensionless Kratky plots (**c**) for the wild type and

G3C mutants of the 11 xrRNAs in the presence of 5 mM EDTA or 5 mM Mg^{2+}. (**d-f**) The scattering curves (**d**)**,** normalized PDDFs (**e**) and the dimensionless Kratky plots (**f**) for the wild type and J2/3 mutants of xrRNA1s from DENV2, ZIKV and WNV in the presence of 5 mM EDTA or 5 mM Mg^{2+}. The PK2 lengths and color codes are indicated on the right. Source data are provided as Source Data file.

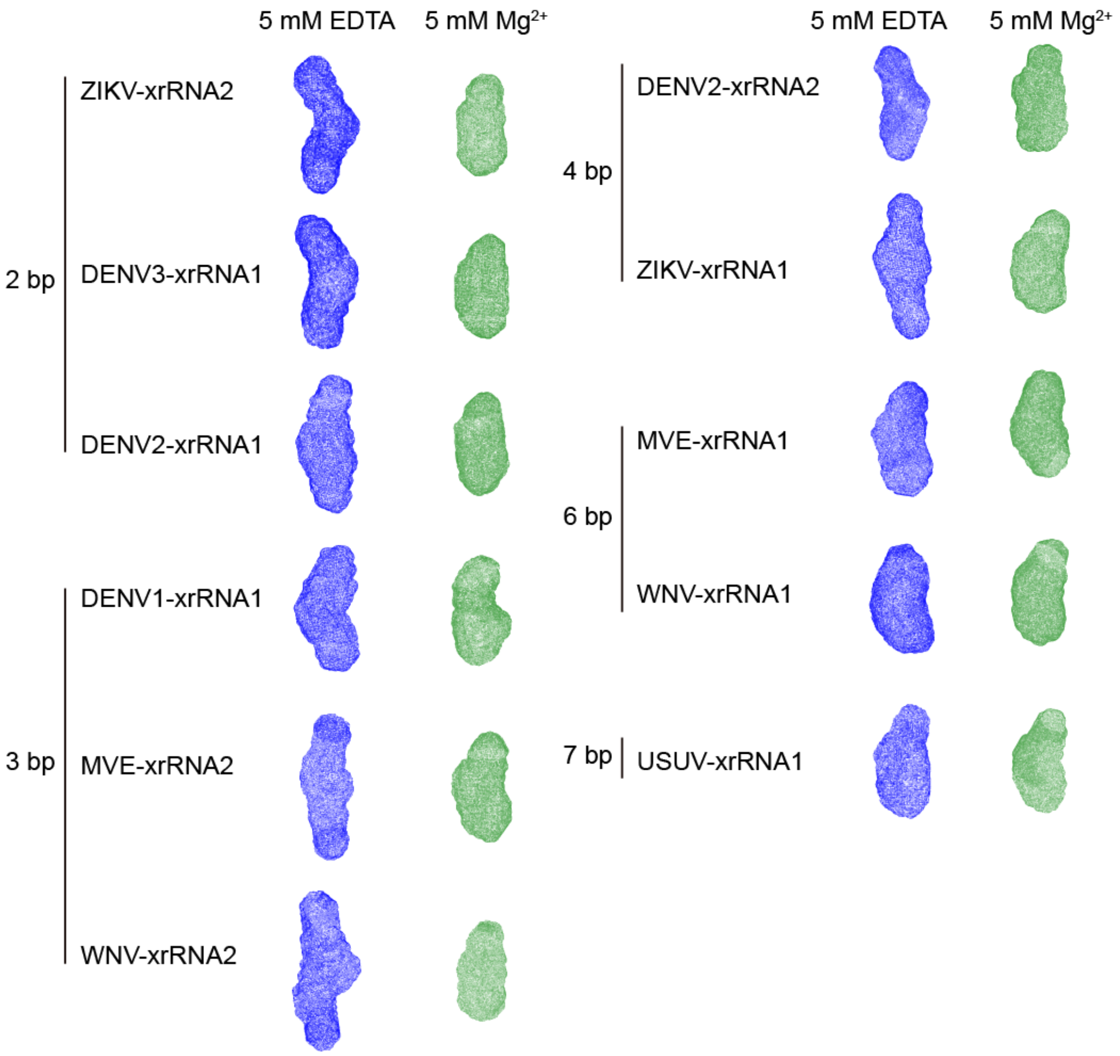

Figure S3. The *ab initio* reconstructed shape envelopes of flaviviral xrRNAs studied in this work in 5 mM EDTA (blue) and 5 mM Mg^{2+} (green). The PK2 lengths are indicated.

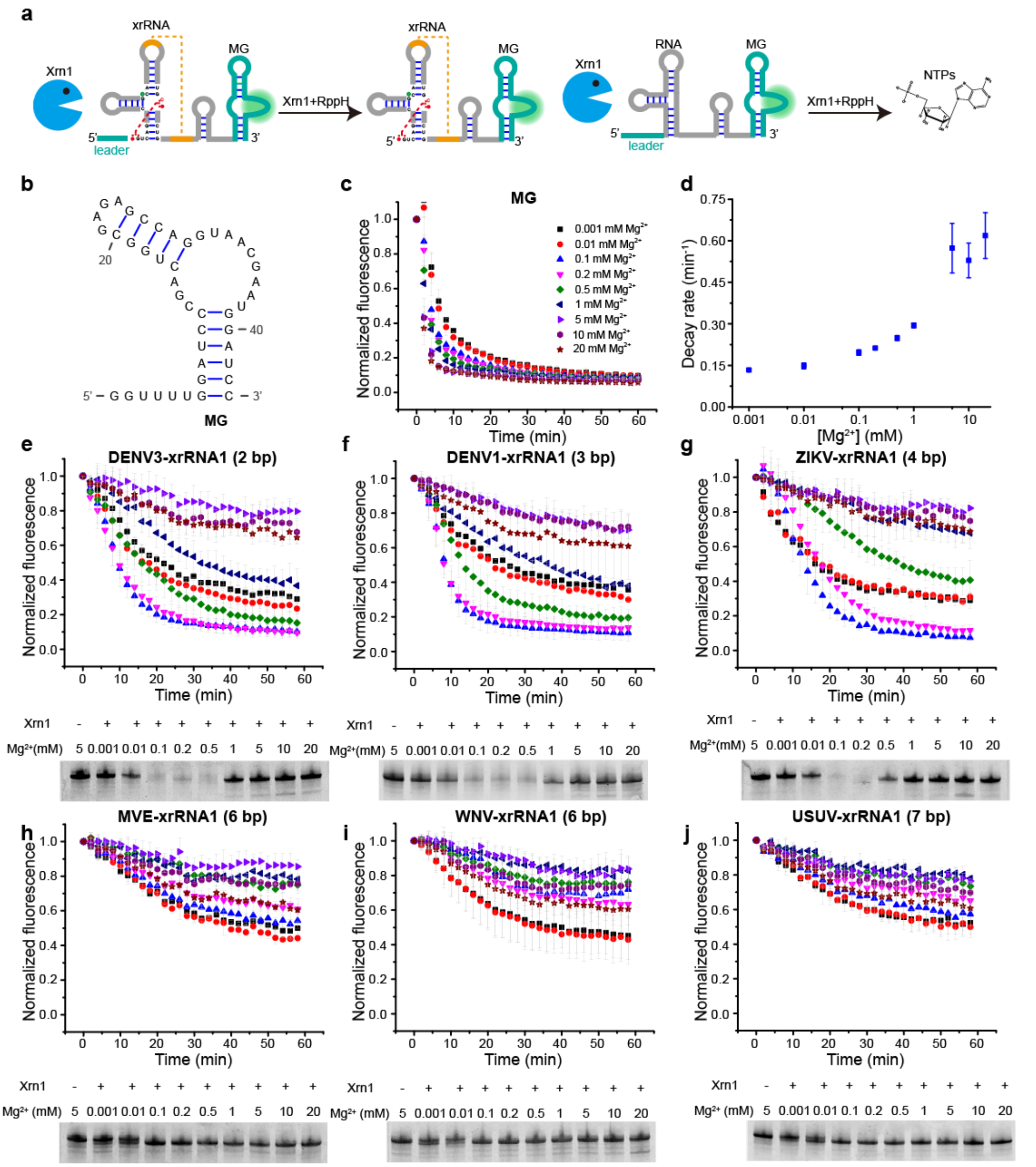

Figure S4. ***In vitro* fluorescence assay for monitoring flaviviral xrRNA1 decay kinetics.** **(a)** Schematic representation of the expected outcome when using Xrn1-resistant (left) or non-resistant (right) RNAs in the fluorescence assay. (**b**) Secondary structure of the malachite green (MG) aptamer sequence used in the Xrn1 decay kinetics assay. (**c**) Normalized fluorescence traces for malachite green (MG) over the course of their reactions with Xrn1 in various Mg^{2+} concentrations (0.001 mM to 20 mM). (**d**) The relative malachite green (MG) decay rates in different Mg^{2+} concentrations. (**e-j**) Normalized fluorescence traces of flaviviral xrRNAs over the course of their reactions with Xrn1 in various Mg^{2+} concentrations. All raw data was normalized by the corresponding values of (-) Xrn1 controls and presented as mean ± SEM. n=3 biologically independent samples examined over 3 independent experiments in **c-j**. The insets are the denaturing PAGE gels showing the Xrn1 decay end products at 60 min. Source data for panels **c-j** are provided as a Source Data file.

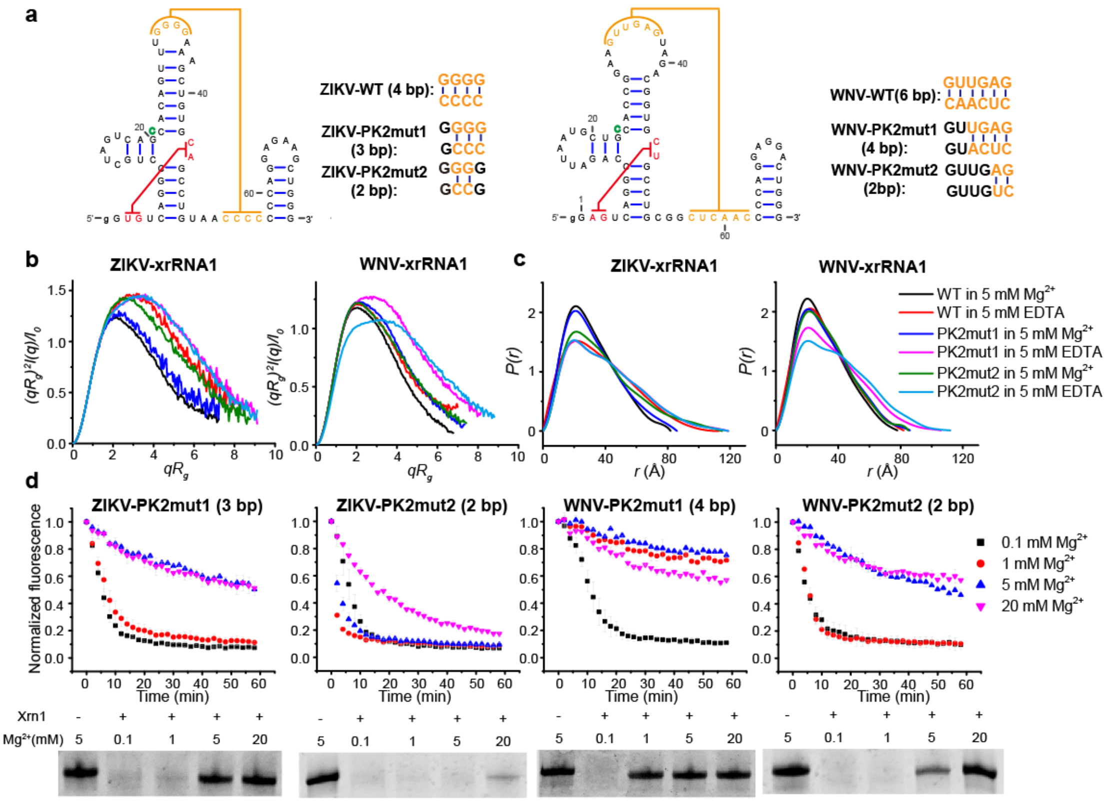

Figure S5. PK2 length variations affect the Mg^{2+}-dependence of folding and Xrn1 resistance of flaviviral xrRNAs. (**a**) Secondary structures of PK2 mutants of ZIKV-xrRNA1 and WNV- xrRNA1. Nucleotides in S4 were mutated to construct the PK2 mutants. (**b-c**) The dimensionless kratky plots (**b**) and normalized PDDFs (**c**) for the PK2 mutants of ZIKV xrRNA1 and WNV xrRNA1 in 5 mM EDTA or Mg^{2+}. (**d**) Xrn1 decay kinetics assay for the PK2 mutants of ZIKV xrRNA1 and WNV xrRNA1 in different Mg^{2+} concentrations. All raw data was normalized by the corresponding values of (-) Xrn1 controls and presented as mean ± SEM. n=3 biologically independent samples examined over 3 independent experiments in **d**. The denaturing PAGE gels show the Xrn1 decay end products at 60 min. Source data for panel b-d are provided as a Source Data file.

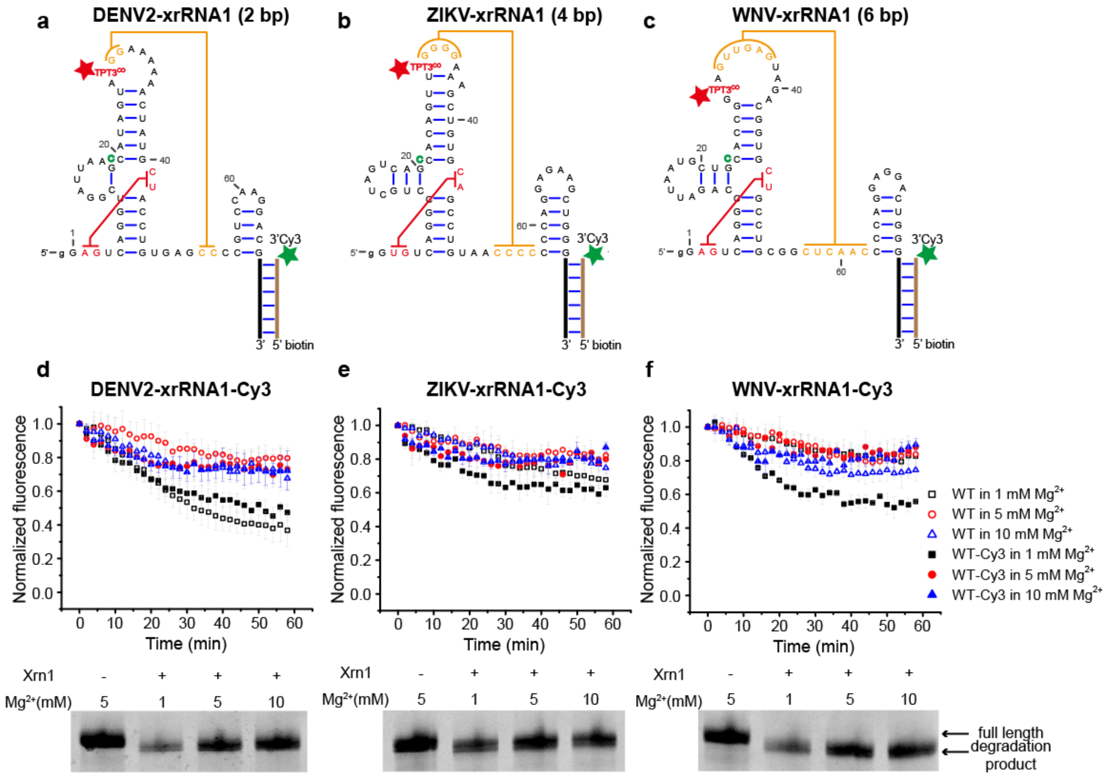

Figure S6. **Fluorescent labeling sites and the effects of fluorescent labeling on the Xrn1 resistance activity of the respective xrRNAs. (a-c)** Labeling sites on the secondary structures of DENV2-xrRNA1 (**a**), ZIKV-xrRNA1 (**b**) and WNV-xrRNA1 (**c**). The Cy5 and Cy3 labeling sites are indicated with red and green stars, respectively. The RNA extension at the 3' end of xrRNAs and the 5'-biotin and 3'-Cy3 labeled DNA oligonucleotides are represented with black and brown lines, respectively. (**d-f**) Xrn1 decay kinetics assay for the Cy3-labeled xrRNAs, showing that the UBP-based site-specific fluorescent labeling has minor effects on the Xrn1 resistance activity of xrRNAs in different Mg^{2+} concentrations. All raw data was normalized by the corresponding values of (-) Xrn1 controls and presented as mean ± SEM. n=3 biologically independent samples examined over 3 independent experiments in **d-f**. The denaturing PAGE gels show the Xrn1 decay end products at 60 min. Source data for panel d-f are provided as a Source Data file.

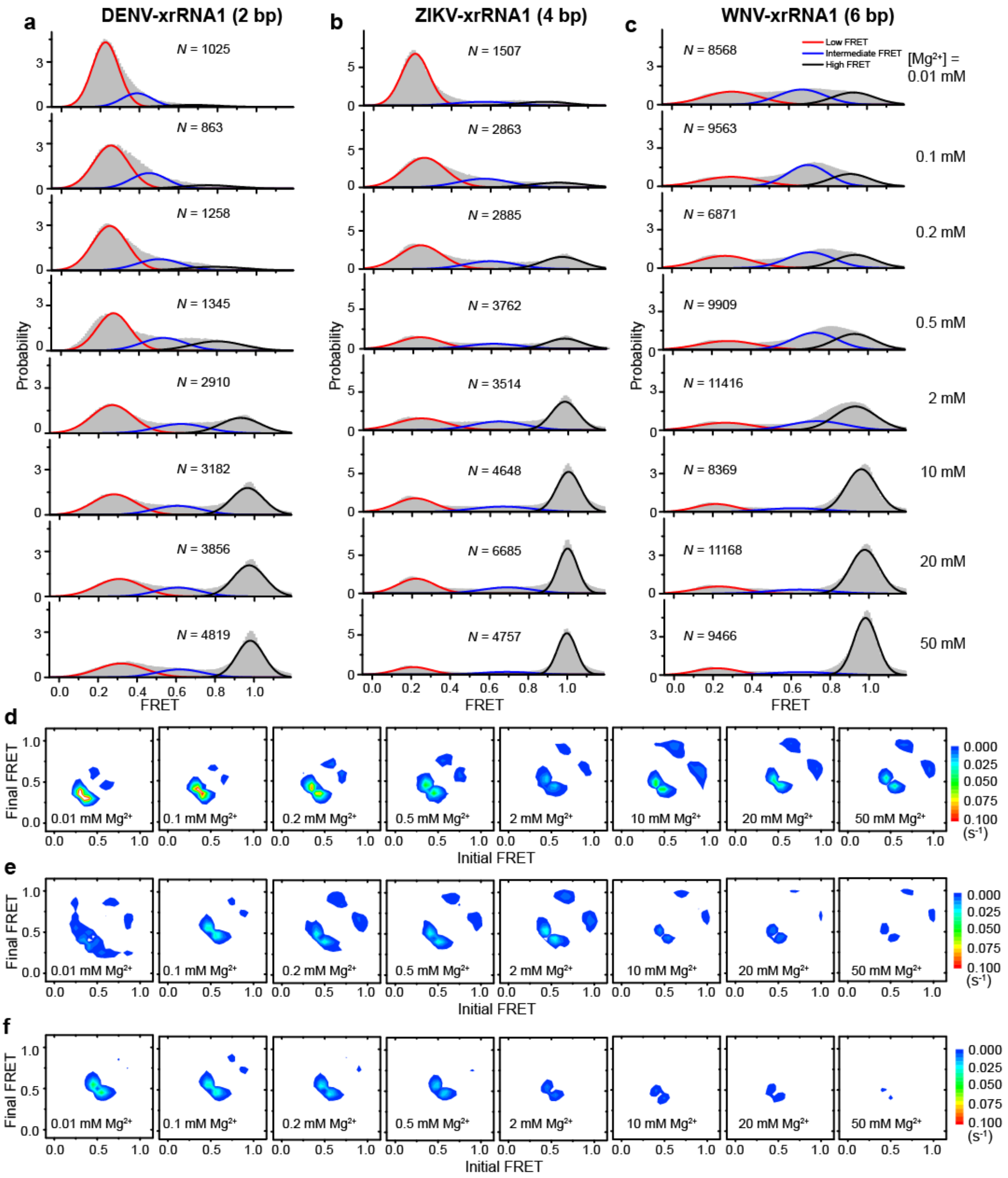

Figure S7. **Mg^{2+}-dependent conformational dynamics of flaviviral xrRNAs by smFRET.** (**a-c**) FRET histograms of DENV2-xrRNA1 (**a**), ZIKV-xrRNA1 (**b**) and WNV-xrRNA1 (**c**) at various Mg^{2+} concentrations ranging from 0.01 mM to 50 mM, which are related to **Figure 3**. *N* denotes the total number of traces used to generate each histogram from three independent experiments. (**d-f**) Transition density plots (TDP) for DENV2-xrRNA1 (**d**), ZIKV-xrRNA1 (**e**) and WNV-xrRNA1 (**f**) at various Mg^{2+} concentrations. TDPs were generated from all smFRET traces from three independent experiments. Source data are provided as a Source Data file.

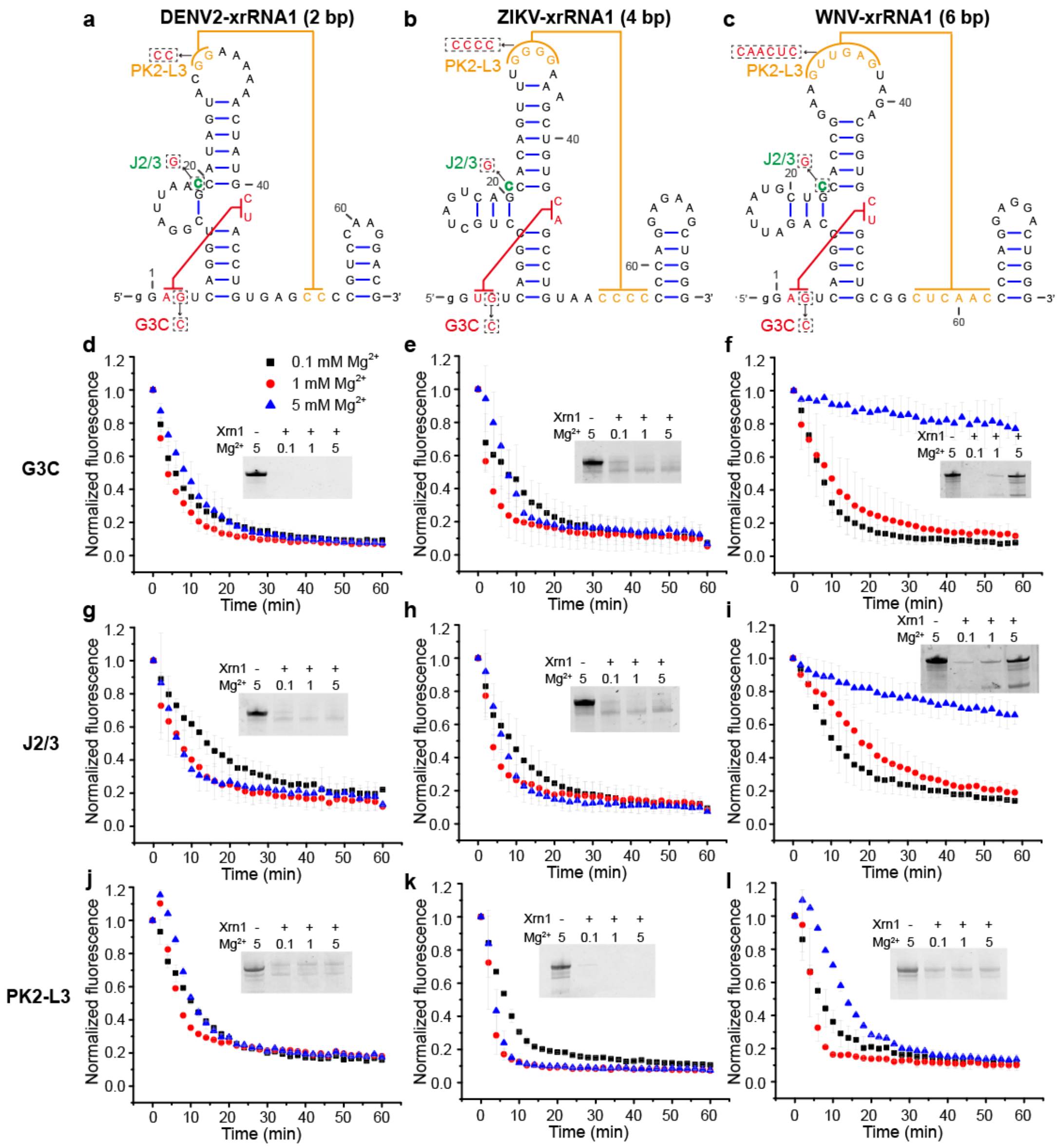

Figure S8. ***In vitro* fluorescence assay for monitoring the decay kinetics of G3C, J2/3 and PK2-L3 mutants of flaviviral xrRNA1s at different Mg^{2+} concentrations. (a-c)** Secondary structure of DENV2-xrRNA1 (**a**), ZIKV-xrRNA1 (**b**) and WNV-xrRNA1 (**c**), the J2/3 and PK2-L3 mutants of all xrRNAs used in this study are indicated with dashed boxes. **(d-l)** The normalized fluorescence traces of the G3C mutants (**d-f**), J2/3 mutants (**g-i**) and PK2-L3 mutants (**j-l**) of DENV2-xrRNA1 (**left**), ZIKV-xrRNA1 (**middle**) and WNV-xrRNA1 (**right**) over the course of their reactions with Xrn1 in various Mg^{2+} concentrations. All raw data was normalized by the corresponding values of (-) Xrn1 controls and presented as mean ± SEM. n=3 biologically independent samples examined over 3 independent experiments in **d-l**. The insets are the denaturing PAGE gel analysis of the Xrn1 decay end products at 60 min. Source data for panels d-l are provided as a Source Data file.

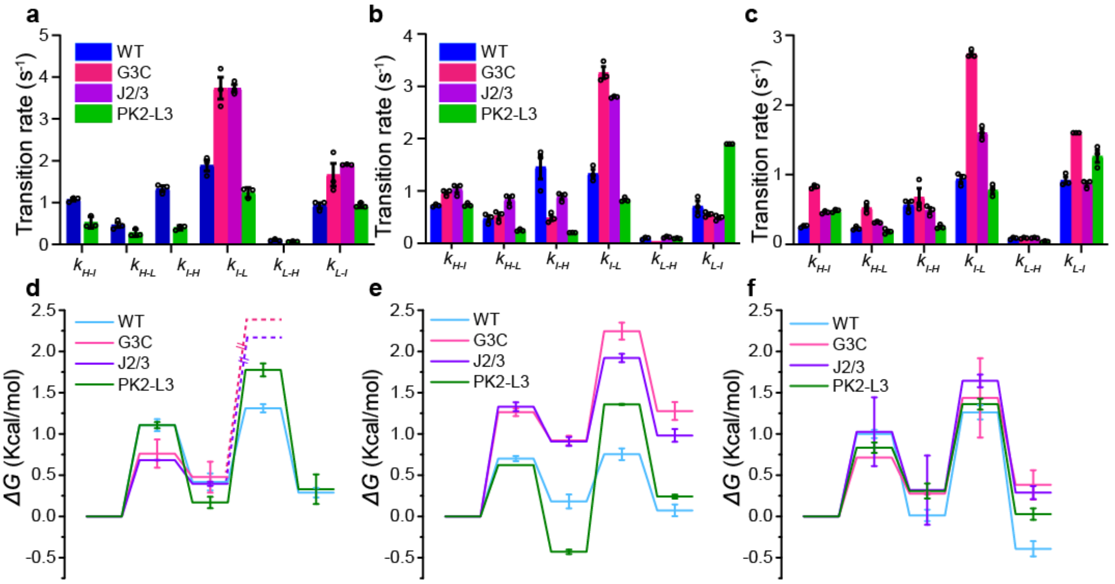

Figure S9. **Mutations at key tertiary interactions affect the dynamics and energy landscape of flaviviral xrRNA.** (**a-c**) Transition rate constants among the L_{state}, I_{state} and H_{state} of the wild type and mutants of DENV2-xrRNA1 (**a**), ZIKV-xrRNA1 (**b**) and WNV-xrRNA1 (**c**) at 10 mM, 5 mM and 5 mM Mg^{2+}, respectively. (**d-f**) Free energy diagrams for the folding of wild type and mutants of DENV2-xrRNA1 (**d**), ZIKV-xrRNA1 (**e**) and WNV-xrRNA1 (**f**) at 10 mM, 5 mM and 5 mM Mg^{2+}, respectively. Data are presented as mean ± SEM. Error bars are SEM of 3 independent experiments (n=3) in **a-f**. Source data are provided as a Source Data file.

Table S1. The primary sequences of RNAs used in this study.

Constructs	RNA sequences
DENV2-xrRNA1	5'GGAGUCAGGUCGGAUUAAGCCAUAGUACGGAAAAAACUAUGCUACCUGUGAGCCCCGUCCAAGGACGU-3'
DENV3-xrRNA1	5'-GGUGUCAGGCCACCUUAAGCCACAGUACGGAAGAAGCUGUGCUGCCUGUGAGCCCCGUCCAAGGACGU-3'
ZIKV-xrRNA2	5'-GAGUCAGGCCGAGAACGCCAUGGCACGGAAGAAGCCAUGCUGCCUGUGAGCCCCUCAGAGGACACUGAGU-3'
DENV1-xrRNA1	5'-GGAGUCAGGCCGGAUUAAGCCAUAGCACGGUAAGAGCUAUGCUGCCUGUGAGCCCCGUCCAAGGACGU-3'
MVE-xrRNA2	5'-GUGUCAGAUCGGCGAAAGUCGCCACUUCGCCGAGGAGUGCAAUCUGUGAGGCCCCAGGAGGACUGGGU-3'
WNV-xrRNA2	5'-GUGUCAGACCACACUUUAAUGUGCCACUCUGCGGAGAGUGCAGUCUGCGAUAGUGCCCCAGGUGGACUGGGU-3'
ZIKV-xrRNA1	5'-GGUGUCAGGCCUGCUAGUCAGCCACAGUUUGGGGAAAGCUGUGCAGCCUGUAACCCCCCCAGGAGAAGCUGGGA -3'
DENV2-xrRNA2	5'GGUCAGGCCAUCAUAAAUGCCAUAGCUUGAGUAAACUAUGCAGCCUGUAGCUCCACCUGAGAAGGUGU-3'
MVE-xrRNA1	5'-GGAGUCAGGCCAGCCGGUUAGGCUGCCACCGAAGGUUGGUAGACGGUGCUGCCUGCGACCAACCCCAGGAGGACUGGGU-3'
WNV-xrRNA1	5'-GGAGUCAGGCCAGAUUAAUGCUGCCACCGGAAGUUGAGUAGACGGUGCUGCCUGCGGCUCAACCCCAGGAGGACUGGGU-3'
USUV-xrRNA1	5'-GGAGUCAGGCCAGGGCAACCUGCCACCGGAAGUUGAGUAGACGGUGCUGCCUGCGACUCAACCCCAGGCGGACUGGGU-3'
Malachite green (MG)	5'GGUUUUGGAUCCCGACUGGCGAGAGCCAGGUAACGAAUGGAUCC-3'

(Note: the constructs used for Xrn1 decay kinetics assay are the above sequences extended with a leader sequence at the 5' end and MG aptamer sequence at the 3' end.)

Table S2. The natural and unnatural oligonucleotide primers used in this study.

Primers	Sequences
pMVF	5'GTAACCCACTCGTGCACCCAACTGATCTTC3'
DENV2-xrRNA1-R	5'CGTCCTTGGACGGGGCTCACAGGTAGCATA3'
DENV3-xrRNA1-R	5'CGTCCTTGGACGGGGCTCACAGGCAGCAC3'
ZIKV-xrRNA2-R	5'CTCAGTGTCCTCTGAGGGGCTCACAGGCAGC3'
DENV1-xrRNA1-R	5'CGTCCTTGGACGGGGCTCACAGGCAGCATA3'
MVE-xrRNA2-R	5'CCCAGTCCTCCTGGGGCCTCACAGATTGCAC3'
WNV-xrRNA2-R	5'CCCAGTCCACCTGGGGCACTATCGCAGACTG3'
ZIKV-xrRNA1-R	5'CCCAGCTTCTCCTGGGGGGGTTACAGGCTGC3'
DENV2-xrRNA2-R	5'CACCTTCTCAGGTGGAGCTACAGGCTGCATAG3'
MVE-xrRNA1-R	5'CCCAGTCCTCCTGGGGTTGGTCGCAGGCAGC3'
WNV-xrRNA1-R	5'CCCAGTCCTCCTGGGGTTGAGCCGCAGGCAG3'
USUV-xrRNA1-R	5'CCCAGTCCGCCTGGGGTTGAGTCGCAGGCAG3'
MG-R	5'GGATCCATTCGTTACCTGGCTCTCGCCAGTCG3'
DENV2-xrRNA1-N27-R	5'CGGGGCTCACAGGTAGCATAGTTTTTCC(**NaM**)TACT3'
DENV2-xrRNA1-linker-R	5'CGGCCTGACTATGGGCTTGGAAACGTCCTTGGACGGGGCTCA3'
ZIKV-xrRNA1-N29-R	5'TGGTCGCAGGCAGCACCGTCTACCAAC(**NaM**)TT3'
ZIKV-xrRNA1-linker-R	5'CGGCCTGACTATGGGCTTGGAACCCAGCTTCTCCTGGGGGGG3'
WNV-xrRNA1-N30-R	5'GCCGCAGGCAGCACCGTCTACTCAACT(**NaM**)CCG3'
WNV-xrRNA1-linker-R	5'CGGCCTGACTATGGGCTTGGAACCCAGTCCTCCTGGGGTTG3'
DNA-linker	5'biotin-CGGCCTGACTATGGGCTTGG-3'Cy3

Table S3. Basic structural parameters for the wild type and mutants of flaviviral xrRNAs in 5 mM Mg^{2+} or 5 mM EDTA by SAXS.

PK2	RNAs		Buffer	$^{a}R_g$ (Å)	$^{b}R_g$ (Å)	D_{max} (Å)	^{c}MW	^{d}MW
2 bp	ZIKV-xrRNA2	WT	Mg^{2+}	24.8±0.3	25.6±0.1	85	21.54	25.96
			EDTA	27.9±0.4	30.1±0.2	105		25.76
		G3C	Mg^{2+}	30.2±0.8	32.5±0.4	115		30.94
			EDTA	31±1	32.9±0.3	119		24.58
	DENV3-xrRNA1	WT	Mg^{2+}	21.7±0.3	22.5±0.1	76	20.82	22.89
			EDTA	26.1±0.4	27.0±0.4	98		24.64
		G3C	Mg^{2+}	29±1	29.9±0.3	110		27.07
			EDTA	32±1	34.3±0.8	120		26.06
	DENV2-xrRNA1	WT	Mg^{2+}	21.9±0.2	22.7±0.1	77	20.86	23.20
			EDTA	28.3±0.8	30.7±0.3	112		25.41
		G3C	Mg^{2+}	27.5±0.5	29.0±0.5	108		22.87
			EDTA	28.6±0.8	30.1±0.7	113		22.67
		C20G	Mg^{2+}	29±1	30.6±0.2	113		30.53
			EDTA	29±1	30.5±0.6	115		25.41
3bp	DENV1-xrRNA1	WT	Mg^{2+}	22.0±0.2	22.5±0.1	78	20.88	24.64
			EDTA	26.0±0.5	27.6±0.3	98		24.30
		G3C	Mg^{2+}	26.1±0.3	27.1±0.4	98		21.48
			EDTA	26.6±0.4	27.6±0.5	105		20.69
	MVE-xrRNA2	WT	Mg^{2+}	24.5±0.7	25.6±0.2	87	20.95	26.75
			EDTA	30.9±0.6	33.0±0.5	117		19.04
		G3C	Mg^{2+}	36±2	38.6±0.2	126		27.25
			EDTA	35±1	37.4±0.3	125		28.57
	WNV-xrRNA2	WT	Mg^{2+}	22.8±0.4	23.5±0.1	79	22.03	24.22
			EDTA	30.4±0.5	32.0±0.6	117		24.57
		G3C	Mg^{2+}	32±1	34.8±0.3	122		27.29
			EDTA	32.7±0.8	34.9±0.6	123		28.5
4bp	DENV2-xrRNA2	WT	Mg^{2+}	22.3±0.2	23.6±0.1	79	20.76	24.48
			EDTA	27.1±0.4	28.1±0.5	106		20.67
		G3C	Mg^{2+}	29.8±1.0	32.0±0.3	116		24.83
			EDTA	30.6±0.9	32.5±0.6	119		22.22
	ZIKV-xrRNA1	WT	Mg^{2+}	23.0±0.4	23.5±0.1	80	22.74	25.47
			EDTA	28.7±0.8	30.2±0.2	113		21.99
		G3C	Mg^{2+}	28.2±0.5	29.6±0.4	110		28.85
			EDTA	29.2±0.5	30.4±0.4	115		22.30
		C22G	Mg^{2+}	27.7±0.4	29.1±0.4	107		29.38
			EDTA	28.5±0.5	30.5±0.4	110		27.14
		PK2mut1	Mg^{2+}	24.4±0.1	25.6±0.4	86		26.30
			EDTA	30.4±0.7	32.5±0.5	119		31.80
		PK2mut2	Mg^{2+}	29.3±0.9	31.2±0.6	115		32.20
			EDTA	30.4±0.7	32.4±0.4	119		31.90
6 bp	MVE-xrRNA1	WT	Mg^{2+}	23.6±0.5	24.4±0.2	83	24.36	23.49
			EDTA	24.6±0.2	25.5±0.2	87		22.68
		G3C	Mg^{2+}	25.4±0.4	27.1±0.1	90		21.12
			EDTA	30.5±0.5	33.4±0.2	118		26.45

	WNV-xrRNA1	WT	Mg^{2+}	23.3±0.2	24.6±0.1	82	24.33	23.46
			EDTA	23.4±0.2	24.1±0.2	82		23.42
		G3C	Mg^{2+}	23.9±0.2	24.7±0.1	84		26.15
			EDTA	30.3±0.5	31.9±0.4	118		29.44
		C24G	Mg^{2+}	23.8±0.3	24.7±0.1	84		26.2
			EDTA	30.4±0.6	31.6±0.7	119		28.52
		PK2mut1	Mg^{2+}	24.5±0.3	25.1±0.1	86		28.50
			EDTA	27.1±0.3	28.7±0.2	106		30.31
		PK2mut2	Mg^{2+}	24.8±0.3	25.2±0.2	85		29.7
			EDTA	29.4±0.5	31.1±0.3	112		33.4
7 bp	USUV-xrRNA1	WT	Mg^{2+}	24.1±0.2	24.8±0.2	86	24.01	21.04
			EDTA	24.6±0.2	25.4±0.3	89		21.61
		G3C	Mg^{2+}	24.1±0.6	24.7±0.1	85		28.32
			EDTA	28.8±0.6	30.8±0.5	113		26.31

[a]derived from Guinier fitting;
[b]derived from GNOM analysis;
[c]MW: molecular weight predicted from sequences;
[d]MW: molecular weight calculated based on the power law of volume of correlation[3];
All of the experiments were conducted in the buffer containing 20 mM Tris (pH 7.5), 100 mM KCl, 5 mM Mg^{2+} or 5 mM EDTA.

Table S4. smFRET efficiencies, populations and transition rates for DENV2-, ZIKV- and WNV- xrRNA1s at different Mg^{2+} concentrations.

RNA	Mg^{2+} (mM)	FRET efficiency [a]			FRET state population (%) [b]			Transition （%）	Transition rate (s^{-1}) [c]					
		L[d]	I[e]	H[f]	L[d]	I[e]	H[f]		k_{H-I}	k_{H-L}	k_{I-H}	k_{I-L}	k_{L-H}	k_{L-I}
DENV2-xrRNA1	0.001	0.23	0.42	0.77	73	22	5	97.6	5.4 ± 1.1	0.7 ± 0.1	0.8 ± 0.2	2.9 ± 0.6	0.03 ± 0.02	0.8 ± 0.1
	0.01	0.23	0.40	0.71	76	19	5	97.7	3.0 ± 0.2	0.75 ± 0.05	0.8 ± 0.1	3.2 ± 0.6	0.03 ± 0.01	0.7 ± 0.1
	0.1	0.26	0.44	0.76	62	29	9	96.5	2.7 ± 0.3	0.7 ± 0.2	0.54 ± 0.09	2.3 ± 0.2	0.04 ± 0.01	1.13 ± 0.06
	0.2	0.25	0.49	0.80	66	22	11	96.2	1.8 ± 0.7	0.52 ± 0.07	0.71 ± 0.04	2.0 ± 0.2	0.054 ± 0.006	0.87 ± 0.06
	0.5	0.25	0.52	0.81	54	24	21	96.1	1.1 ± 0.2	0.43 ± 0.03	0.93 ± 0.25	1.9 ± 0.1	0.10 ± 0.02	0.9 ± 0.3
	1	0.25	0.57	0.87	53	22	24	95.2	1.29 ± 0.09	0.46 ± 0.05	1.3 ± 0.2	2.1 ± 0.1	0.09 ± 0.02	0.77 ± 0.06
	2	0.27	0.62	0.94	51	20	28	94.1	1.17 ± 0.08	0.46 ± 0.07	1.08 ± 0.12	2.03 ± 0.14	0.10 ± 0.02	0.73 ± 0.14
	5	0.27	0.58	0.94	42	19	39	94.3	1.19 ± 0.09	0.39 ± 0.03	1.6 ± 0.1	2.2 ± 0.1	0.083 ± 0.006	0.8 ± 0.0
	10	0.28	0.61	0.97	39	19	41	93.0	1.07 ± 0.06	0.5 ± 0.1	1.3 ± 0.1	1.9 ± 0.2	0.10 ± 0.02	0.9 ± 0.1
	20	0.31	0.60	0.99	35	18	47	93.2	0.88 ± 0.06	0.39 ± 0.06	1.57 ± 0.02	1.9 ± 0.3	0.13 ± 0.02	0.9 ± 0.1
	50	0.32	0.62	0.99	31	17	51	94.0	0.67 ± 0.07	0.30 ± 0.03	1.6 ± 0.1	1.95 ± 0.08	0.16 ± 0.04	0.9 ± 0.2
	100	0.32	0.62	0.99	27	18	55	94.1	0.58 ± 0.03	0.24 ± 0.02	1.5 ± 0.1	2.01 ± 0.07	0.14 ± 0.02	1.0 ± 0.1
ZIKV-xrRNA1	0.001	0.24	0.52	0.89	74	16	11	96.5	2.1 ± 0.5	1.3 ± 0.2	1.2 ± 0.3	3.1 ± 0.1	0.18 ± 0.03	0.65 ± 0.08
	0.01	0.22	0.45	0.82	75	12	14	96.6	1.9 ± 0.6	1.7 ± 0.3	1.7 ± 0.3	3.2 ± 0.1	0.23 ± 0.04	0.42 ± 0.03
	0.1	0.27	0.57	0.99	64	23	13	95.4	1.53 ± 0.05	0.7 ± 0.2	0.8 ± 0.2	2.3 ± 0.5	0.07 ± 0.02	0.9 ± 0.1
	0.2	0.25	0.60	0.98	53	21	27	93.1	1.4 ± 0.2	0.76 ± 0.04	1.5 ± 0.3	2.0 ± 0.2	0.20 ± 0.04	1.1 ± 0.4
	0.5	0.26	0.62	1.02	44	23	33	91.0	1.34 ± 0.09	0.81 ± 0.06	1.2 ± 0.1	1.7 ± 0.1	0.14 ± 0.08	1.3 ± 0.6
	1	0.28	0.64	0.99	41	27	32	93.3	1.30 ± 0.06	0.59 ± 0.04	1.1 ± 0.1	1.6 ± 0.4	0.12 ± 0.02	1.1 ± 0.2
	2	0.26	0.65	1.00	32	24	45	91.8	1.14 ± 0.02	0.53 ± 0.03	1.9 ± 0.9	1.9 ± 0.7	0.147 ± 0.006	1.4 ± 0.1
	5	0.25	0.67	1.04	35	19	46	90.7	0.87 ± 0.03	0.58 ± 0.04	1.2 ± 0.3	1.5 ± 0.4	0.09 ± 0.02	0.81 ± 0.08

	10	0.25	0.68	1.03	30	17	53	91.3	0.72 ± 0.03	0.47 ± 0.09	1.5 ± 0.4	1.3 ± 0.1	0.09 ± 0.02	0.7 ± 0.2
	20	0.24	0.66	1.04	29	16	55	90.9	0.65 ± 0.02	0.44 ± 0.02	1.03 ± 0.06	1.3 ± 0.1	0.09 ± 0.02	0.8 ± 0.2
	50	0.24	0.67	1.02	24	12	64	91.8	0.50 ± 0.07	0.29 ± 0.01	1.7 ± 0.1	1.9 ± 0.1	0.08 ± 0.04	0.7 ± 0.2
	100	0.24	0.65	1.00	23	12	66	94.1	0.47 ± 0.06	0.20 ± 0.04	2.31 ± 0.09	1.93 ± 0.07	0.06 ± 0.02	0.6 ± 0.1
WNV-xrRNA1	0.001	0.32	0.64	0.98	41	37	22	94.6	0.55 ± 0.04	0.25 ± 0.01	0.36 ± 0.05	1.4 ± 0.1	0.031 ± 0.008	1.3 ± 0.2
	0.01	0.31	0.66	0.96	37	36	27	95.3	0.45 ± 0.06	0.25 ± 0.03	0.29 ± 0.02	1.09 ± 0.08	0.033 ± 0.006	1.3 ± 0.1
	0.1	0.31	0.69	0.95	28	44	27	95.3	0.51 ± 0.04	0.24 ± 0.02	0.28 ± 0.01	0.773 ± 0.006	0.05 ± 0.02	1.27 ± 0.06
	0.2	0.29	0.7	0.98	34	37	29	93.7	0.6 ± 0.1	0.30 ± 0.03	0.35 ± 0.05	0.9 ± 0.1	0.05 ± 0.00	1.15 ± 0.01
	0.5	0.30	0.71	0.96	26	40	34	95.0	0.45 ± 0.06	0.24 ± 0.02	0.32 ± 0.03	0.71 ± 0.03	0.057 ± 0.006	1.18 ± 0.09
	1	0.27	0.69	0.90	21	22	57	95.2	0.30 ± 0.02	0.28 ± 0.01	0.42 ± 0.05	0.8 ± 0.1	0.11 ± 0.02	0.98 ± 0.09
	2	0.26	0.72	0.98	22	29	49	93.9	0.29 ± 0.02	0.22 ± 0.02	0.40 ± 0.03	0.73 ± 0.02	0.087 ± 0.006	1.05 ± 0.06
	5	0.26	0.69	0.96	19	17	64	94.1	0.26 ± 0.02	0.23 ± 0.03	0.57 ± 0.09	0.94 ± 0.09	0.09 ± 0.02	0.92 ± 0.09
	10	0.24	0.62	0.97	17	12	71	94.1	0.27 ± 0.02	0.247 ± 0.006	0.8 ± 0.1	1.3 ± 0.2	0.07 ± 0.02	0.983 ± 0.006
	20	0.25	0.64	1.00	17	12	70	92.8	0.21 ± 0.04	0.23 ± 0.05	0.69 ± 0.02	1.1 ± 0.3	0.08 ± 0.01	0.94 ± 0.06
	50	0.24	0.63	1.00	15	9	76	93.9	0.19 ± 0.02	0.17 ± 0.02	0.96 ± 0.09	1.23 ± 0.05	0.06 ± 0.01	0.81 ± 0.09
	100	0.24	0.61	1.00	14	9	77	93.4	0.20 ± 0.03	0.17 ± 0.02	1.2 ± 0.7	1.7 ± 0.9	0.07 ± 0.01	0.9 ± 0.1

All of the experiments were conducted in the buffer containing 50 mM HEPES (pH 7.5), 100 mM KCl and 5 mM or 10 mM Mg^{2+}.
All results were averages of three independent experiments. [a] SEM (standard error of the mean) is 0.01 or lower; [b] SEM is 1% or lower; [c] SEM is listed. [d] Low FRET; [e] Intermediate FRET; [f] High FRET.

Table S5. smFRET efficiencies, populations and transition rates of DENV2-, ZIKV- and WNV- xrRNA1 mutants at high Mg^{2+}.

RNA	Mutants	FRET efficiency [a]			FRET state population (%) [b]			Transition (%)	Transition rate (s^{-1}) [c]					
		L[d]	I[e]	H[f]	L[d]	I[e]	H[f]		$k_{H\text{-}I}$	$k_{H\text{-}L}$	$k_{I\text{-}H}$	$k_{I\text{-}L}$	$k_{L\text{-}H}$	$k_{L\text{-}I}$
DENV2-xrRNA1 (10 mM Mg^{2+})	G3C	0.30	0.60	-	81	19	-	98.0	-	-	-	2.6 ± 0.2	-	1.0 ± 0.1
	C20G	0.31	0.58	-	75	25	-	97.9	-	-	-	2.6 ± 0.2	-	1.8 ± 0.2
	PK2-L3	0.27	0.46	0.78	26	31	43	96.4	0.50 ± 0.05	0.22 ± 0.02	0.43 ± 0.02	1.27 ± 0.06	0.06 ± 0.01	0.9 ± 0.2
ZIKV-xrRNA1 (5 mM Mg^{2+})	G3C	0.26	0.47	0.87	83	13	4	97.3	1.0 ± 0.2	0.5 ± 0.1	0.49 ± 0.01	3.24 ± 0.08	0.031 ± 0.003	0.51 ± 0.05
	C20G	0.25	0.56	1.02	75	14	12	95.3	1.0 ± 0.1	0.9 ± 0.1	0.69 ± 0.08	2.3 ± 0.3	0.12 ± 0.02	0.49 ± 0.06
	PK2-L3	0.30	0.63	0.88	28	60	13	97.2	0.70 ± 0.08	0.23 ± 0.03	0.197 ± 0.006	0.84 ± 0.03	0.09 ± 0.02	1.9 ± 0.4
WNV-xrRNA1 (5 mM Mg^{2+})	G3C	0.35	0.61	0.95	48	32	20	96.6	0.54 ± 0.01	0.38 ± 0.01	0.30 ± 0.02	2.0 ± 0.1	0.06 ± 0.01	1.2 ± 0.2
	C24G	0.31	0.66	0.96	47	23	30	94.9	0.469 ± 0.007	0.324 ± 0.005	0.49 ± 0.03	1.6 ± 0.1	0.09 ± 0.02	0.9 ± 0.2
	PK2-L3	0.32	0.74	0.93	32	43	26	95.1	0.40 ± 0.06	0.19 ± 0.03	0.3 ± 0.1	0.93 ± 0.03	0.36 ± 0.06	1.0 ± 0.2

All of the experiments were conducted in the buffer containing 50 mM HEPES (pH 7.5), 100 mM KCl and 5 mM or 10 mM Mg^{2+}.
All results were averages of three independent experiments. [a] SEM (standard error of the mean) is 0.01 or lower; [b] SEM is 1% or lower; [c] SEM is listed. [d] Low FRET; [e] Intermediate FRET; [f] High FRET.

References

1. Akiyama BM*, et al.* Zika virus produces noncoding RNAs using a multi-pseudoknot structure that confounds a cellular exonuclease. *Science* **354**, 1148-1152 (2016).
2. Zhang Y*, et al.* Long non-coding subgenomic flavivirus RNAs have extended 3D structures and are flexible in solution. *EMBO reports* **20**, e47016 (2019).
3. Rambo RP, Tainer JA. Accurate assessment of mass, models and resolution by small-angle scattering. *Nature* **496**, 477-481 (2013).
