# Supplementary material for: Pseudoknot length modulates the folding, conformational dynamics, and robustness of Xrn1 resistance of flaviviral xrRNAs
Source: Nat Commun. 2021 Nov 5;12:6417. doi: 10.1038/s41467-021-26616-x (PMC8571300; doi:10.1038/s41467-021-26616-x)
Supplement: Supplementary file 3 — Description of Additional Supplementary Files [file 41467_2021_26616_MOESM3_ESM.pdf]

Description of Additional Supplementary Files

File Name: Supplementary Data 1
Description: Custom code for smFRET data analysis
