# Supplementary material for: Pseudoknot length modulates the folding, conformational dynamics, and robustness of Xrn1 resistance of flaviviral xrRNAs
Source: Nat Commun. 2021 Nov 5;12:6417. doi: 10.1038/s41467-021-26616-x (PMC8571300; doi:10.1038/s41467-021-26616-x)
Supplement: Supplementary file 5 — Reporting Summary [file 41467_2021_26616_MOESM5_ESM.pdf]

nature research

Corresponding author(s): Chunlai Chen, Xianyang Fang

Last updated by author(s): Sep 27, 2021

Reporting Summary

Nature Research wishes to improve the reproducibility of the work that we publish. This form provides structure for consistency and transparency in reporting. For further information on Nature Research policies, see our Editorial Policies and the Editorial Policy Checklist.

Statistics

For all statistical analyses, confirm that the following items are present in the figure legend, table legend, main text, or Methods section.

n/a	Confirmed	
☐	☒	The exact sample size (*n*) for each experimental group/condition, given as a discrete number and unit of measurement
☐	☒	A statement on whether measurements were taken from distinct samples or whether the same sample was measured repeatedly
☒	☐	The statistical test(s) used AND whether they are one- or two-sided<br>*Only common tests should be described solely by name; describe more complex techniques in the Methods section.*
☒	☐	A description of all covariates tested
☒	☐	A description of any assumptions or corrections, such as tests of normality and adjustment for multiple comparisons
☐	☒	A full description of the statistical parameters including central tendency (e.g. means) or other basic estimates (e.g. regression coefficient) AND variation (e.g. standard deviation) or associated estimates of uncertainty (e.g. confidence intervals)
☒	☐	For null hypothesis testing, the test statistic (e.g. *F*, *t*, *r*) with confidence intervals, effect sizes, degrees of freedom and *P* value noted<br>*Give P values as exact values whenever suitable.*
☒	☐	For Bayesian analysis, information on the choice of priors and Markov chain Monte Carlo settings
☒	☐	For hierarchical and complex designs, identification of the appropriate level for tests and full reporting of outcomes
☐	☒	Estimates of effect sizes (e.g. Cohen's *d*, Pearson's *r*), indicating how they were calculated

Our web collection on statistics for biologists contains articles on many of the points above.

Software and code

Policy information about availability of computer code

Data collection	Cell Vision software (Beijing Coolight Technology) for smFRET data collection;
Data analysis	HaMMy, ImageJ 1.43u plugin (http://rsb.info.nih.gov/ij), MatlabR2017a and OriginPro2016 for smFRET data analysis; MatlabR2017a, BioXTAS RAW1.3.1, PRIMUS3.2, GNOM4.6, CRYSOL2.8.3, PyMol1.8 for SAXS data analysis; ModeRNA (http://iimcb.genesilico.pl/modernaserver/) for RNA homologous structure modeling; Origin2016 for Xrn1 decay kinetics data analysis.

For manuscripts utilizing custom algorithms or software that are central to the research but not yet described in published literature, software must be made available to editors and reviewers. We strongly encourage code deposition in a community repository (e.g. GitHub). See the Nature Research guidelines for submitting code & software for further information.

Data

Policy information about availability of data

All manuscripts must include a data availability statement. This statement should provide the following information, where applicable:

- Accession codes, unique identifiers, or web links for publicly available datasets
- A list of figures that have associated raw data
- A description of any restrictions on data availability

The data supporting the findings of this study are available from the corresponding authors upon reasonable request. The source data underlying Figs.1c, 1f, 3a-m, 4a-k, 5a, 5e-g, 6a-i and supplementary fig. S2a-f, S4c-j, S5b-d, S6d-f, S7a-f, S8d-l and S9a-f are provided as a Source Data file with this paper. Custom codes used to analyze smFRET data are provided with this paper.

Field-specific reporting

Please select the one below that is the best fit for your research. If you are not sure, read the appropriate sections before making your selection.

☒ Life sciences ☐ Behavioural & social sciences ☐ Ecological, evolutionary & environmental sciences

For a reference copy of the document with all sections, see nature.com/documents/nr-reporting-summary-flat.pdf

Life sciences study design

All studies must disclose on these points even when the disclosure is negative.

Sample size	Sample size for smFRET experiment is the number of molecules as described in methods. Experiments were repeated for 3 times and N > 800 molecules were selected for analysis. And in general, for most conditions, about 3000 to 8000 molecules were analyzed, which is sufficient for statistical analysis.
Data exclusions	No data were excluded from the analysis.
Replication	The results were successfully reproduced in at least 3 independent experiments.
Randomization	Samples were not allocated into experimental groups, thus randomization is not relevant for our study.
Blinding	There was no group allocation during data collection and analysis. So blinding is not necessary.

Reporting for specific materials, systems and methods

We require information from authors about some types of materials, experimental systems and methods used in many studies. Here, indicate whether each material, system or method listed is relevant to your study. If you are not sure if a list item applies to your research, read the appropriate section before selecting a response.

Materials & experimental systems

n/a	Involved in the study
☒	☐ Antibodies
☒	☐ Eukaryotic cell lines
☒	☐ Palaeontology and archaeology
☒	☐ Animals and other organisms
☒	☐ Human research participants
☒	☐ Clinical data
☒	☐ Dual use research of concern

Methods

n/a	Involved in the study
☒	☐ ChIP-seq
☒	☐ Flow cytometry
☒	☐ MRI-based neuroimaging
